# Cadherin repeat 5 mutation associated with Bt resistance in a field-derived strain of pink bollworm

**DOI:** 10.1038/s41598-020-74102-z

**Published:** 2020-10-08

**Authors:** Ling Wang, Yuemin Ma, Wei Wei, Peng Wan, Kaiyu Liu, Min Xu, Shengbo Cong, Jintao Wang, Dong Xu, Yutao Xiao, Xianchun Li, Bruce E. Tabashnik, Kongming Wu

**Affiliations:** 1 Key Laboratory of Integrated Pest Management on Crops in Central China, Ministry of Agriculture, Hubei Key Laboratory of Crop Disease, Insect Pests and Weeds Control, Institute of Plant Protection and Soil Fertility, Hubei Academy of Agricultural Sciences, Wuhan, 430064 China; 2 State Key Laboratory for Biology of Plant Diseases and Insect Pests, Institute of Plant Protection, Chinese Academy of Agricultural Sciences, Beijing, 100193 China; 3 School of Life Science, Central China Normal University, Wuhan, 430079 China; 4 Agricultural Genomics Institute at Shenzhen, Chinese Academy of Agricultural Sciences, Shenzhen, 518120 China; 5 Department of Entomology, University of Arizona, Tucson, AZ 85721 USA

**Keywords:** Biological techniques, Microbiology

## Abstract

Evolution of resistance by pests reduces the benefits of transgenic crops that produce insecticidal proteins from *Bacillus thuringiensis* (Bt). Here we analyzed resistance to Bt toxin Cry1Ac in a field-derived strain of pink bollworm (*Pectinophora gossypiella*), a global pest of cotton. We discovered that the *r14* allele of the pink bollworm cadherin gene (*PgCad1*) has a 234-bp insertion in exon 12 encoding a mutant PgCad1 protein that lacks 36 amino acids in cadherin repeat 5 (CR5). A strain homozygous for this allele had 237-fold resistance to Cry1Ac, 1.8-fold cross-resistance to Cry2Ab, and developed from neonate to adult on Bt cotton producing Cry1Ac. Inheritance of resistance to Cry1Ac was recessive and tightly linked with *r14*. *PgCad1* transcript abundance in midgut tissues did not differ between resistant and susceptible larvae. Toxicity of Cry1Ac to transformed insect cells was lower for cells expressing *r14* than for cells expressing wild-type *PgCad1.* Wild-type PgCad1 was transported to the cell membrane, whereas PgCad1 produced by *r14* was not. In larval midgut tissue, PgCad1 protein occurred primarily on the brush border membrane only in susceptible larvae. The results imply *r14* mediates pink bollworm resistance to Cry1Ac by reduced translation, increased degradation, and/or mislocalization of cadherin.

## Introduction

Transgenic crops producing insecticidal proteins from *Bacillus thuringiensis* (Bt) are useful for controlling some of the world’s most destructive pests^1–4^. First commercialized in 1996, Bt crops have been planted on a cumulative total of more than 1 billion hectares worldwide during the past 25 years^4^. Bt crops kill some major target pests, yet are harmless to most non-target organisms, yielding considerable economic and environmental benefits^1–9^. However, evolution of pest resistance to Bt crops has reduced these benefits^10–13^.

Advances in understanding the mechanisms of pest resistance are needed to better monitor, manage, and counter pest resistance to Bt crops. To kill insects, Bt toxins must bind to receptor proteins in the larval midgut^14^. Reduced toxin binding is one of the most efficient and common mechanisms of insect resistance to Bt toxins^15–18^. Reduced toxin binding can be caused by mutations that alter the amino acid sequence of receptor proteins or reduce the concentration of these proteins, such as cadherins, ATP-binding cassette (ABC) transporter proteins, aminopeptidases N, and alkaline phosphatases^15–18^. Resistance to Bt toxins in the Cry1A subfamily is often associated with changes in the sequence or transcription of midgut cadherins in at least five species of lepidopteran pests^15,16,18^.

Here we focus on resistance to Bt toxin Cry1Ac associated with a novel allele (*r14*) of the cadherin gene *PgCad1* in the pink bollworm, *Pectinophora gossypiella*, a global cotton pest^19,20^. Pink bollworm is one of nine major pests that have evolved practical resistance to Bt crops, which is defined as field-evolved resistance that has practical implications for pest management^11–13^. In India, the pink bollworm rapidly evolved practical resistance to Cry1Ac and Cry2Ab produced by transgenic cotton^13^. By contrast, sustained susceptibility to Bt cotton producing these toxins was essential for the recent declaration of eradication of pink bollworm from the continental United States^13^. Also, populations in China remain susceptible to the Cry1Ac-producing cotton grown there^13,21,22^. These different outcomes are consistent with the idea that refuges of non-Bt cotton delayed resistance in China and the United States, but were scarce or absent in India^13^.

Previous work has identified pink bollworm resistance to Bt toxin Cry1Ac associated with 17 mutant alleles of *PgCad1* that disrupt the amino acid sequence of the encoded protein (*r1–r16*) or reduce its transcription (*r17*)^22–29^. We previously reported isolation of *PgCad1* allele *r14* from a field-collected individual from Anqing in the Yangtze River Valley of China^26^, but the mutation site and resistance mechanism of *r14* were not known. In this study, we discovered that *r14* has a 234-bp insertion in exon 12 predicted to cause the absence of 36 amino acids in the cadherin repeat 5 (CR5) of PgCad1 protein. We also found that this mutation is tightly linked with recessive, 237-fold resistance to Cry1Ac, but not with reduced transcription of *PgCad1*. The results imply *r14* mediates pink bollworm resistance to Cry1Ac by reduced translation, increased degradation, and/or mislocalization of cadherin.

## Results

### Sequencing of *PgCad1* allele *r14* and allele-specific PCR

**Figure 1:**
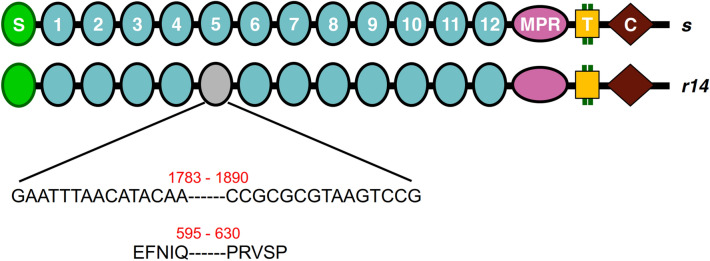
Schematic representation of the predicted PgCad1 protein in pink bollworm. The amino-terminal membrane signal sequence (S), cadherin repeats (1–12), membrane proximal region (MPR), transmembrane region (T), and cytoplasmic domain (C) are shown for the wild type (*s*, at top). Red numbers indicate deletions in the cDNA (108 bp) and 36 amino acids missing from the protein potentially encoded by the mutant allele *r14* (bottom).

Sequencing of *PgCad1* cDNA revealed the *r14* allele from Cry1Ac-resistant strain AQ189 has a deletion of 108 bases (1783 to 1890) (Fig. 1), encoding a PgCad1 protein that lacks 36 amino acids in CR5, which corresponds with the amino acids encoded by exon 12 (Figs. 1 and S1). Genomic DNA sequencing showed that this deletion is associated with a 234-bp insertion in exon 12 (Fig. S2).

Based on the 234 bp insertion in *r14* gDNA, we designed a pair of allele-specific primers to identify *r14*. These primers produce a single band of 852 bp in individuals homozygous or heterozygous for *r14* (*r14r14* or *r14s)*, but no band in individuals lacking *r14* (*ss)* (Fig. S3a, n = 30 individuals per genotype). We also designed another pair of primers to distinguish between *r14r14* and *r14s*. These primers produce no band for *r14r14,* but a single band of 853 bp for *r14s* (Fig. S3b, n = 30 individuals per genotype)*.*

### Inheritance of resistance to Cry1Ac and cross-resistance to Cry2Ab

Based on LC_50_ values, AQ189 had 237-fold resistance to Cry1Ac relative to APHIS-S (Table 1). No significant difference occurred in the LC_50_ of Cry1Ac between the F_1_ progeny from the two reciprocal crosses of AQ189 and APHIS-S (Table 1), implying autosomal inheritance. At 10 μg Cry1Ac per mL diet, larval survival was 89% for AQ189 and 0% for APHIS-S and the F_1_ progeny from crosses between APHIS-S and AQ189 (n = 72 larvae per treatment). This yields a value of 0 for the dominance parameter *h* and indicates resistance was inherited recessively at this concentration.

**Table 1:** Toxicity of Cry1Ac to pink bollworm larvae from resistant strain AQ189, susceptible strain APHIS-S, and their hybrid F_1_ progeny.

Strain	Slope (SE)^a^	LC_50_ (95% FL)^b^	RR^c^
APHIS-S	2.9 (0.3)	0.104 (0.089–0.12)	
AQ189	3.0 (0.6)	24.6 (20–30)	237
AQ189♀ × APHIS-S♂	2.0 (0.2)	0.446 (0.22–0.80)	4.3
AQ189♂ × APHIS-S♀	2.5 (0.3)	0.632 (0.42–0.94)	6.1

^a^Slope of the concentration-mortality line with its standard error in parentheses.

^b^Concentration killing 50% with 95% fiducial limits in parentheses, in μg Cry1Ac per mL diet.

^c^Resistance ratio, the LC_50_ for AQ189, AQ189♀ × APHIS-S♂ or AQ189♂ × APHIS-S♀ divided by the LC_50_ for APHIS-S.

The LC_50_ of Cry2Ab (in μg Cry2Ab per mL diet) was 0.286 (95% FL: 0.23 – 0.34) for AQ189 versus 0.157 (95% FL: 0.12 – 0.19) for APHIS-S (Table S1). The 1.8-fold resistance to Cry2Ab in AQ189 relative to APHIS-S is statistically significant based on the lack of overlap between the 95% FL of the LC_50_ values.

### Genetic linkage analysis

To determine if resistance to Cry1Ac in AQ189 is linked with *r14,* we conducted a genetic linkage analysis. We established five backcross families by pairing a single female from AQ189 with a single male F_1_ (from AQ189 × APHIS-S) for each of five pairs. On control diet, 75 survivors were *r14s* and 79 were *14r14*, which does not differ significantly from the expected ratio of 1:1 (one-sample t-test, t = -0.52, df = 4, P = 0.63, Table S2). On diet with 10 μg Cry1Ac per mL diet, all survivors were *r14r14* (n = 100, 20 from each backcross family, Table S2). On diet treated with Cry1Ac, the proportion of survivors with the *r14r14* genotype was significantly higher than on untreated diet (Fisher’s exact test, P < 0.00001). These results demonstrate resistance to Cry1Ac in AQ189 was tightly linked with *r14*.

### Life History Traits of AQ189 and APHIS-S on Cotton Bolls

On bolls of Bt cotton, larvae survival was significantly higher for AQ189 (13.9%) than APHIS-S (0.0%) (t-test, t = 7.9, df = 4, P = 0.0014, Table S3). On bolls of non-Bt cotton, larval survival did not differ significantly between AQ189 (28.5%) and APHIS-S (31.1%) (t-test, t = 1.5, df = 4, P = 0.20, Table S3). Also, on non-Bt cotton bolls, pupal weight did not differ significantly between these two strains (Table 2). However, the time to pupation on non-Bt cotton was significantly longer for AQ189 (16.3 days) than APHIS-S (15.0 days) (Table 2). The slower development on non-Bt cotton for AQ189 relative to APHIS-S implies a fitness cost associated with resistance affected this trait in AQ189.

**Table 2:** Time to pupation and pupal weight for pink bollworm reared on Bt and non-Bt cotton bolls.

Strain	Cotton type	Number of pupae	Time to pupation (days)	Pupal wt. (mg)
APHIS-S	Non-Bt	70	15.0 ± 0.2 a	13.7 ± 0.4 a
AQ189	Non-Bt	30	16.3 ± 0.4 b	13.3 ± 0.6 a
AQ189	Bt	16	21.1 ± 0.8 c	11.3 ± 0.7 b

Values are means ± SE. Different lower case letters within columns indicate significant differences between treatments based on ANOVA followed by Tukey’s HSD.

For AQ189, survival from neonate to adult and the proportion of adults that were female were lower on bolls of Bt cotton than non-Bt cotton (Fisher’s exact test, P < 0.00001 for each trait). In addition, AQ189 development was significantly slower on Bt cotton than non-Bt cotton (Table 2). Also, the number of eggs laid per female was lower for females that developed on bolls of Bt cotton than non-Bt cotton (t = 5.0, df = 4, P = 0.007). However, the percentage of eggs hatching did not differ significantly for females from bolls of Bt cotton versus non-Bt cotton (t = 0.19, df = 6.8, *P* = 0.82) (Table 3). The net reproductive rate for AQ189 was 4.3 times higher on non-Bt cotton (12.9) than Bt cotton (3.0) (Table 3), implying incomplete resistance of AQ189 to Bt cotton.

**Table 3:** Life history traits of resistant pink bollworm strain AQ189 reared on Bt and non-Bt cotton bolls^a^.

Trait	Bt	Non-Bt	Bt/non-Bt
Neonate to adult survival^b^	0.14 (112)	0.29 (129)	0.48
Proportion of females	0.33 (15)	0.43 (28)	0.77
Eggs per female	80 ± 3 (5)	126 ± 9 (12)	0.63
Hatch rate^c^	0.80 ± 0.03 (169)	0.82 ± 0.04 (707)	0.98
Net reproductive rate^d^	3.0	12.9	0.23

^a^Sample sizes are shown in parentheses.

^b^Proportion of neonates that became adults = the number of adults divided by the number of entry holes, which reflects the number of neonates that entered cotton bolls (112 for Bt and 129 for non-Bt).

^c^Proportion of eggs that hatch = the number of eggs that hatch divided by the total number of eggs, values are means plus or minus standard errors.

^d^Neonate to adult survival × proportion of females × eggs per female × hatch rate.

### Localization and Transcript Abundance of PgCad1 in Larval Midgut Tissues of AQ189 and APHIS-S

Based on analysis of midgut tissue sections of fourth instar larvae using immunofluorescence, PgCad1 protein occurred mainly on the brush border of the epithelial cells from APHIS-S, but not AQ189 (Fig. 4). We also determined the relative transcript abundance of *PgCad1* in midgut tissues of fourth instar larvae using fluorescence quantitative real-time PCR. The mean (± SE) relative *PgCad1* transcript abundance did not differ significantly between AQ189 (0.89 ± 0.11) and APHIS-S (1.00 ± 0.031) (Fig. S4, t-test, df = 6, t =  − 0.92, P = 0.39).

### PgCad1 Localization and Susceptibility to Cry1Ac in Transfected Cells

**Figure 2:**
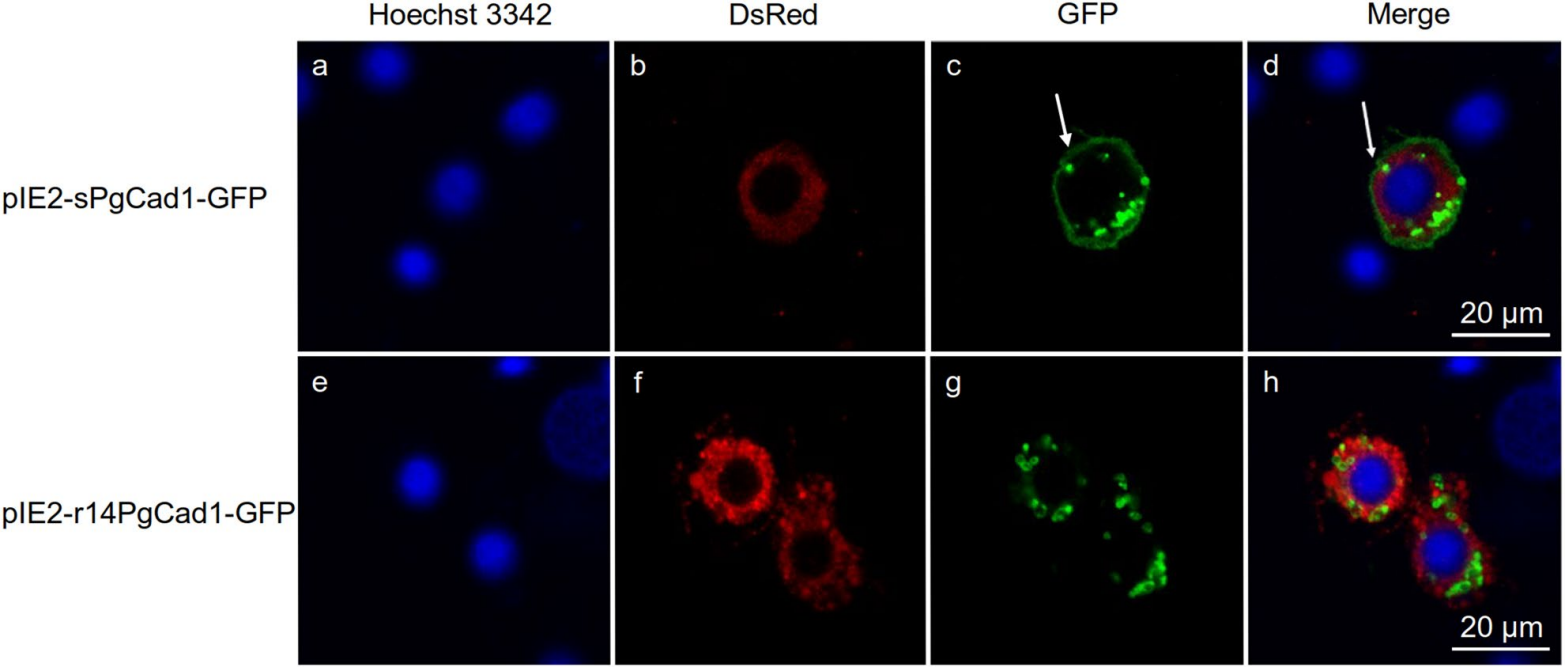
Cellular localization of PgCad1 protein within Hi5 cells. Hi5 cells transfected with pIE2-sPgCad1-GFP (**a–d**) and pIE2-r14PgCad1-GFP (**e**–**h**). Nuclei stained with Hoechst 3342 are shown in blue, DsRED-labeled endoplasmic reticulum in red, and GFP-labeled PgCad1 fusion proteins in green. Superimposed images from (**a–c**) are shown in (**d**) and from (**e**–**g**) in (**h**). The arrow in (**c**) and (**d**) indicates the cell membrane.

We transfected the recombinant plasmids containing the open reading frame of either the *s* or *r14* allele into Hi5 cells to generate the PgCad1-GFP fusion proteins sPgCad1-GFP or r14PgCad1-GFP, respectively (Fig. 2). Transfection efficiency (mean % ± SE) did not differ significantly between sPgCad1-GFP (69 ± 9%) and r14PgCad1-GFP (60 ± 7%) (t-test, t = 0.75, df = 4, *P* = 0.50). Western blotting demonstrated the molecular weight of fusion protein sPgCad1-GFP (253 kDa) and r14PgCad1-GFP (248 kDa) expressed by transfected Hi5 cells was consistent with the expected weight (Fig. S5). However, the band representing recombinant r14PgCad1-GFP was much weaker than the band for recombinant sPgCad1-GFP (Fig. S5).

The pIE2-DsRed2-ER plasmid that generated an endoplasmic reticulum (ER) tag protein with red fluorescence was simultaneously transfected into Hi5 cells expressing PgCad1-GFP fusion protein (Fig. 2). The fusion protein sPgCad1-GFP occurred primarily with the cell membrane (Fig. 2d), whereas r14PgCad1-GFP occurred together with the ER (Fig. 2h). These results show that *r14* was associated with mislocalization of cadherin in transfected cells.

**Figure 3:**
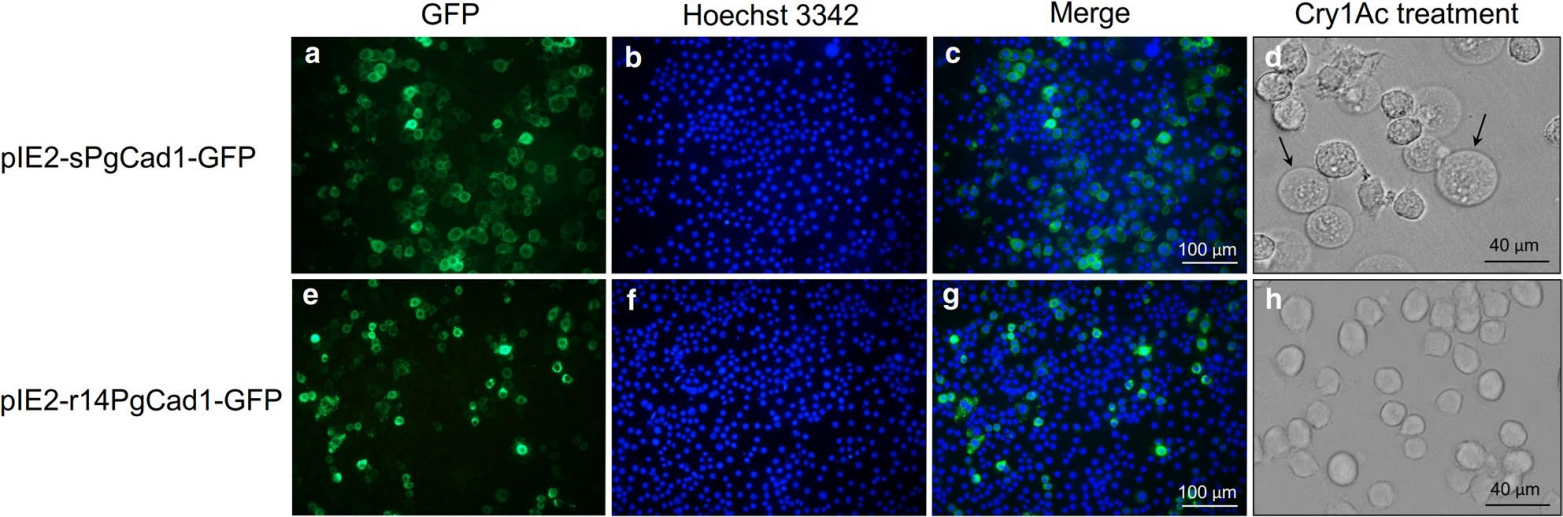
Toxic effects of Cry1Ac on transformed Hi5 cells producing PgCad1 fusion proteins. Hi5 cells were transfected with pIE2-sPgCad1-GFP (**a–d**) or pIE2-r14PgCad1-GFP (**e**–**h**). Cells in (**d**) and (**h**) were treated with 10 or 40 μg Cry1Ac per mL, respectively, and observed for swelling using fluorescence microscopy. Arrows in (**d**) point to representative swollen cells. Cells in (**a–c**) and (**e–g**) are untreated controls, all shown at the same magnification. For these controls, GFP-labeled PgCad1 fusion proteins are green and nuclei stained with Hoechst 3342 are blue. Superimposed images from (**a–b**) are shown in (**c**), and from (**e**–**f**) in (**g**).

After treatment with Cry1Ac, Hi5 cells producing sPgCad1-GFP were swollen and lysed (Fig. 3a–d), while the cells producing r14PgCad1-GFP (Fig. 3e–h) showed normal morphology. For the cells producing sPgCad1-GFP, the concentration of Cry1Ac causing half of the cells to swell was 7.3 μg per mL (95% FL: 6.2 to 8.4). By contrast, no swelling occurred in the cells producing r14PgCad1-GFP at 40 μg Cry1Ac per mL, the highest concentration tested.

## Discussion

Here we analyzed a mutant cadherin allele, *PgCad1* allele *r14*, which is associated with pink bollworm resistance to Cry1Ac and survival on transgenic Bt cotton producing Cry1Ac. The *r14* allele has a 234-bp insertion in exon 12 and producing a transcript with a 108-bp deletion causing the loss of 36 amino acids in the CR5 domain of PgCad1 protein. Because the deletion corresponds exactly with the amino acids encoded by exon 12, we infer the insertion probably causes mis-splicing of exon 12. This mutation differs from those reported previously in 16 other *PgCad1* alleles associated with resistance to Bt toxin Cry1Ac^23–28^.

The binding sites for Cry1A toxins in lepidopteran cadherin are mainly in the CRs adjacent to the membrane proximal region (MPR)^30–34^. However, CR5 (amino acids 595–630), which is disrupted by the mutation in *r14,* is upstream of the reported binding sites for Cry1Ac in PgCad1, including CR9-CR12 and the MPR^33^. Nonetheless, the mutation in *r14* encoding the partial deletion of CR5 was tightly linked with resistance. Like the deletions in *r14*, the deletions in PgCad1 encoded by the *r1* and *r13* alleles are upstream of the toxin-binding regions^23,33^. Although we did not measure binding in this study, we suspect that *r14* reduces binding of Cry1Ac to PgCad1, as observed with other mutations disrupting PgCad1^35^.

In experiments with transfected insect cells, the mislocalization of cadherin associated with *r14* (Fig. 2) is similar to previous results for alleles *r13*, *r15B* and *r16* of pink bollworm and the *mHaCad* allele of *H. armigera*^26–28,36^. Whereas the *r14* mutation disrupts CR5, the mutations in *r13*, *r15B* and *r16* of pink bollworm affect the transmembrane region of PgCad1^26–28^ and *mHaCad* alters the N-terminal region of HaCad^36^. Collectively, these results suggest that correct expression and localization of PgCad1 may rely on several domains of this protein.

In larval midgut tissues, transcript abundance of *PgCad1* did not differ between AQ189 harboring *r14* and the susceptible strain APHIS-S. This is similar to results with the *mHaCad* allele^36^, but differs from the significantly reduced abundance of *PgCad1* transcript associated with the *r17* and *r2* alleles of pink bollworm^29^. For the three other alleles associated with mislocalization of cadherin in pink bollworm (*r13*, *r15B* and *r16*), the relative transcript abundance of *PgCad1* remains to be evaluated.

**Figure 4:**
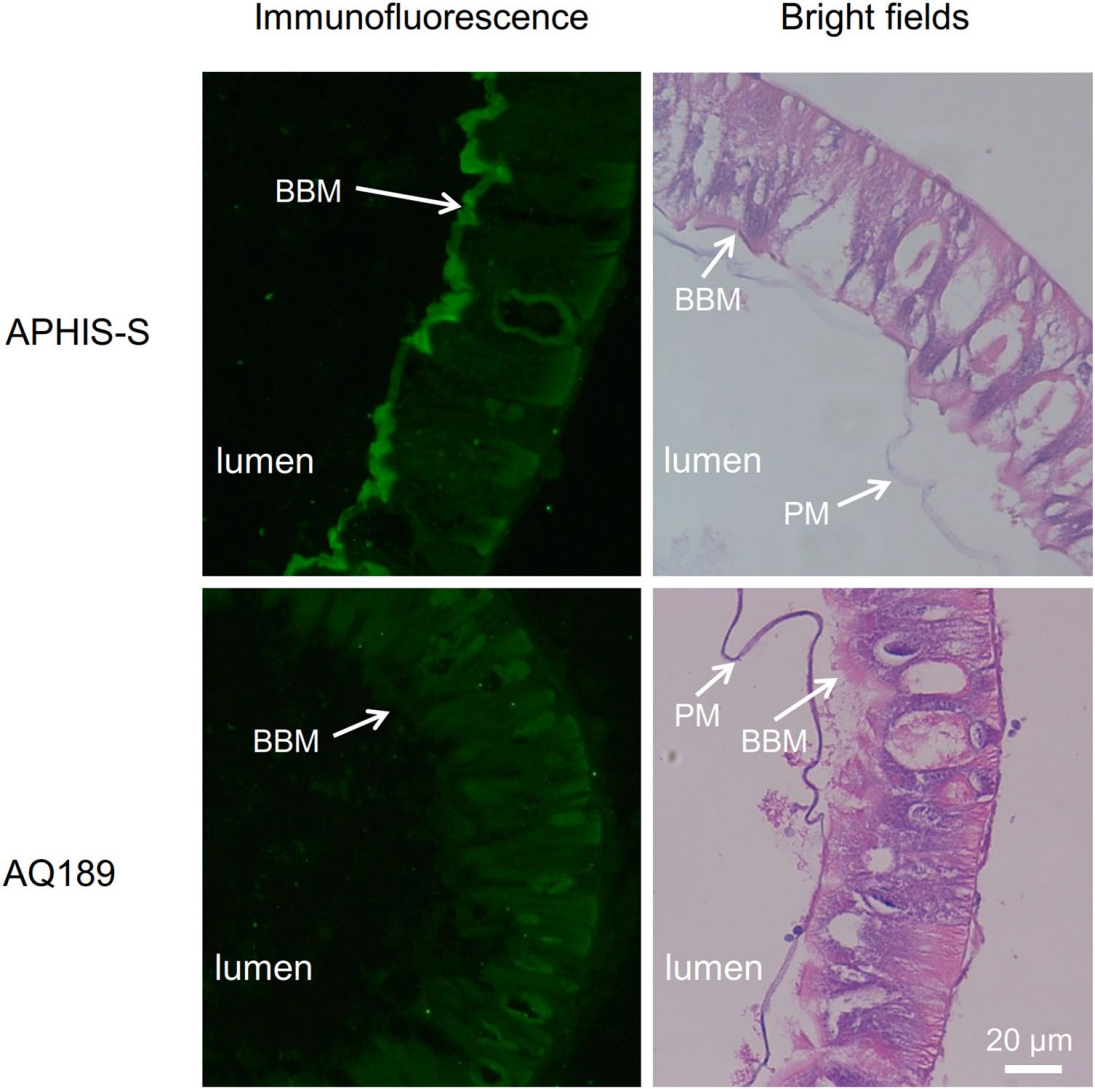
Localization of PgCad1 protein in middle midgut tissue sections of fourth instar larvae of APHIS-S (susceptible) and AQ189 (resistant). PgCad1 protein was revealed by immunofluorescence using rabbit anti-PgCad1 antibody. BBM: brush border membrane. PM: peritrophic membrane.

The transcript abundance of *PgCad1* was not significantly lower in AQ189 than APHIS-S, but the amount of *PgCad1* protein appears to be reduced in AQ189 relative to APHIS-S based on the decreased immunofluorescence in larval midgut cells (Fig. 4) and the fainter band in the Western blot of recombinant cadherin proteins within Hi5 cells (Fig. S5). These results imply that factors contributing to the resistance in AQ189 relative to APHIS-S could include reduced translation of *PgCad1* transcripts, increased degradation of PgCad1 protein, or both.

Although the mutation site and mechanism of *r14* are novel, the resistance phenotype in AQ189 is similar to that of other Cry1Ac-resistant strains of pink bollworm from the U.S. and China harboring mutant *PgCad1* alleles^23,24,26–28,37,38^. Like AQ189, the other strains have > 100-fold resistance to Cry1Ac that is recessively inherited, whereas their cross-resistance to Cry2Ab is weak or nil. Unlike susceptible strains, the resistant strains completed their life cycle on Cry1Ac-producing cotton. However, their performance on Cry1Ac cotton was not as good as on non-Bt cotton, indicating incomplete resistance. On Bt cotton relative to non-Bt cotton, the net reproductive rate was 0.23 for AQ189, which is similar to resistant strains from China harboring *r13* (0.16) and *r15* (0.25) and to U.S. resistant strains carrying alleles *r1, r2* and *r3* (0.35)^26,28,37^.

Including the new results reported here, pink bollworm resistance to Cry1Ac is associated with mutations that affect the amino sequence or quantity of PgCad1 in all lab-selected strains and field-selected populations that have been evaluated^23–29^. Thus, this study and previous results indicate PgCad1 plays a key role in the mode of action of Cry1Ac. Disruption or reduced abundance of this protein can confer high levels of pink bollworm resistance to Cry1Ac.

In DNA screening of field populations of pink bollworm that had sustained susceptibility to Cry1Ac, alleles *r1, r2* and *r3* were not detected in over 9,000 individuals sampled in the southwestern U.S. from 2000 to 2011, and the combined frequency of seven resistance alleles (*r1-r3* and *r13-r16*) was 0.005 in 19,748 individuals sampled in China’s Yangtze River Valley from 2012 to 2015^22,39,40^. The frequency of *r14* was 0.0002^22^. In field-selected, Cry1Ac-resistant populations from India, alleles *r1–r4* identified from lab-selected strains in the U.S. were not detected in 436 individuals screened by PCR, but sequencing of DNA from eight individuals revealed eight novel, severely disrupted *PgCad1* alleles (*r5–r12*) and 19 transcript isoforms generated by mis-splicing^25^. Whereas the low frequency of mutant *PgCad1* alleles in the U.S. and China was associated with sustained susceptibility to Cry1Ac, the high frequency of such alleles in India was associated with practical resistance to Cry1Ac observed there^22,25,39,40^. These results suggest that tracking the frequency of mutant *PgCad1* alleles may be useful for monitoring resistance to Cry1Ac in field populations of pink bollworm. However, given the diversity of resistance alleles and transcript isoforms detected already for *PgCad1*, and the likelihood that even more diversity occurs in field-selected populations, comprehensive monitoring will require detection of mutations throughout this gene.

## Materials and methods

### Insect strains

We used three strains of pink bollworm: a susceptible strain named APHIS-S and two resistant strains named AZP-R and AQ189. The APHIS-S strain, which originated from Arizona, USA, has been reared in the laboratory for over 30 years without exposure to Bt proteins or other insecticides^41^. The AZP-R strain originated from Cry1Ac-resistant individuals collected in 1997 from ten field populations in Arizona and was repeatedly selected with Cry1Ac in the laboratory^23,24,42^. The AQ189 strain, which is composed of homozygous *r14r14* individuals, was isolated from the F_1_ progeny of a single-pair cross between a field-collected male (#47) from Anqing and a female (*PgCad1* genotype *r1r1*) from AZP-R^26^. We generated AQ189 by screening with Cry1Ac, additional single-pair crosses, and DNA screening of *PgCad1* (Fig. S6).

For all three strains, larvae were reared on an artificial diet^43^ and adults supplied with 8% honey solution at 29 ± 1 °C, photoperiod (16L: 8D), relative humidity 50 ± 10% for larvae and 70 ± 10% for adults. Larvae of AQ189 and AZP-R were reared every fifth generation on a diet containing 10 μg Cry1Ac protoxin per mL of diet to maintain resistance.

### Bt toxins

For exposure of larvae to Bt proteins in diet, we used the protoxin form of Cry1Ac from Bt strain HD73 and Cry2Ab from Bt strain B-Pr-88 from Zhongbao Biotechnology Company, Beijing, China. For assays with transformed cells, we used activated Cry1Ac toxin from Marianne Pusztai-Carey (Case Western Reserve University, Cleveland, OH)^26,44^. For activated Cry1Ac, the protoxin inclusion bodies were obtained from *E. coli* cells transformed with the *cry1Ac* gene from the HD1 strain of Bt subsp. *kurstaki*, then solubilized, activated by trypsin, purified, and lyophilized.

### Cloning and sequencing of *PgCad1* from AQ189

Fourth instar larvae of AQ189 strain that survived on diet containing 10 μg Cry1Ac per mL diet were used to clone and sequence the complete cDNA and partial gDNA of *PgCad1* (GenBank accession nos. KY814706 and KY814707.1, respectively). The TaKaRa MiniBEST Universal RNA Extraction Kit (TaKaRa) was used to extract total RNA and gDNA simultaneously from fourth instar larvae (n = 8). We used M-MLV Reverse Transcriptase (Promega) to synthesize the first cDNA strand, and attained the full-length cDNA sequence of *PgCad1* based on PCR amplification using two pairs of specific primers (F1/R1, F2/R2, Table S4). To determine the gDNA flanking sequences of the mutation site from the *r14* allele, PCR amplification was performed using the primers gF189 + gR189 (Table S4) and Ex-Taq DNA polymerase (TaKaRa). The PCR steps were as follows: 98 °C for 1 min, then 33 cycles including 98 °C for 10 s, 58 °C for 30 s and 72 °C for 2 min, and finally 72 °C for 10 min. Subsequently, the PCR products were purified, cloned and sequenced, and the gDNA and cDNA sequences of *r14* were analyzed as reported previously^26^.

### Bioassays

We performed diet bioassays to analyze larval susceptibility of AQ189 to Cry1Ac and Cry2Ab protoxins, compared to the susceptible APHIS-S strain. The concentration of Cry1Ac varied from 0 µg/mL diet (control group) to 10 µg/mL diet for the susceptible strain and the F_1_ progeny from the two reciprocal crosses between APHIS-S and AQ189, and to 40 µg/mL diet for AQ189. The concentration of Cry2Ab varied from 0 µg/mL diet to 1.92 µg/mL diet for APHIS-S and 2.16 µg/mL diet for AQ189. For each concentration, three replicates of 24 neonates each (n = 72) were performed.

### Inheritance of resistance

We established two reciprocal mass crosses by pairing 30 female APHIS-S with 30 male AQ189, and 30 female AQ189 with 30 male APHIS-S in two different 2 L containers to obtain F_1_ offspring. We performed diet bioassays using newly hatched larvae from the two F_1_ sets of progeny as described above. We calculated the dominance parameter *h* ranging from 0 for complete recessive to 1 for complete dominance based on adjusted survival at 10 μg Cry1Ac per mL diet^45^.

### Genetic linkage analysis

We carried out genetic linkage analysis to determine if Cry1Ac resistance in AQ189 is genetically linked with the *r14* allele. We established five backcross families by pairing an F_1_ male (AQ189 APHIS-S) with a female from AQ189 for each family. Crossing over in Lepidoptera occurs only in males^46^. So, to determine if resistance was tightly linked with *r14,* we chose F_1_ males to set up backcross families. For each backcross family, the number of neonates used for bioassays was 90–100, including roughly 40 neonates on control diet and 50–60 neonates on diet treated diet with 10 μg Cry1Ac per mL diet. We performed DNA-based PCR detection using specific primers as mentioned above to genotype all the survivors on the untreated diet or treated diet from each backcross family. We identified genotypes for a total of 254 individuals, with 154 individuals from untreated diet (n = 30, 31, 31, 30, and 32 larvae per backcross family) and 100 individuals from treated diet (n = 20 individuals for each family).

### Life history traits

We determined the life history traits for larvae from AQ189 and APHIS-S on bolls collected from Bt cotton generating Cry1Ac (GK19) and bolls from non-Bt cotton (Simian-3). Both GK19 and Simian-3 were provided by Institute of Cotton Research of CAAS, China. We conducted three replicates with 7–12 bolls from Bt cotton and 6–15 bolls from non-Bt cotton for each strain and replicate. We used 1170 newly hatched larvae including 210 larvae from AQ189 and 350 larvae from APHIS-S on Bt bolls, and 180 larvae from AQ189 and 430 larvae from APHIS-S on non-Bt bolls. The life history characteristics including the number of entry holes, exit holes, pupae, male and female adults, eggs laid by each female, time to pupation, pupal weight, and percentage of eggs hatching were recorded and analyzed as before^26–28^.

### Construction and expression of fusion proteins and Hi5 cell transfection

We amplified the *PgCad1* genes from larval midguts of APHIS-S and AQ189 using a pair of primers (PgCADF and PgCADR) including *Eco*RI and *Sac*II restriction sites (Table S4). The complete open reading frames of *s* and *r14* allele were individually obtained and cloned into the pIE2-EGFP-N1 plasmid, which contains the green fluorescent protein (GFP) gene as a marker^47^. The pIE2-EGFP-N1 plasmid was kindly provided by Dr. Kaiyu Liu, Central China Normal University, China. Recombinant vectors were identified by double enzyme digestion and DNA sequencing and then applied to transfect Hi5 cells (the *Trichoplusia ni* cell line, BTI-Tn-5B1-4 cells^48^) to express the fusion proteins sPgCad1-GFP and r14PgCad1-GFP. We used the pDsRed2-ER vector to label the endoplasmic reticulum (ER) of Hi5 cells, carried out cell transfection, and calculated the transfection efficiency as described previously^26,36^.

To transfect Hi5 cells, we applied 2 μg of the recombinant vector pIE2-sPgCad1-GFP or pIE2-r14PgCad1-GFP. The transformed cells were cultured, lysed, and subsequently analyzed based on immunoblotting. For lysed cells from each sample, the total protein contents were measured by applying Pierce BCA protein assay kit (Thermo). Cell lysates with the same amount (30 µg) of total protein from each sample were separated on a 12% SDS-PAGE gel for 60 min at 70 V and 120 min at 120 V, then transferred to the polyvinylidene fluoride (PVDF) membrane (5 h at 135 mA) and subsequently subjected to immunodetection with anti-GFP (1:4000) and anti-actin (1:4000) monoclonal antibodies and Daylight 800 labeled goat anti-mouse antibody (1:8000) in sequential order as shown previously^26^.

### Cry1Ac toxicity to Hi5 cells

After transfection for 24 h, we tested Cry1Ac toxicity to Hi5 cells transfected with recombinant vectors pIE2-sPgCad1-GFP or pIE2-r14PgCad1-GFP. We detected the transformed cells using a fluorescence microscope after treatment with activated Cry1Ac (six groups ranging from 0 to 40 μg per mL PBS) for one hour. We evaluated cell toxicity using the ratio of swollen cells as described previously^47^. We performed three repeats for each group and applied the ratio of swollen cells for six observed visual fields per repeat per group to determine cells toxicity.

### PgCad1 detection in midgut tissue sections using immunofluorescence

We dissected fourth instar larvae from AQ189 and APHIS-S to obtain their midgut tissue and immediately applied 4% paraformaldehyde for fixing the tissues. The dehydrated midgut tissue was wrapped in paraffin and sliced into sections via a microtome as previously described^49^. Next, we fixed midgut tissue sections on salinized glass slides to dewax and rehydrate, and applied 10 mM citrate buffer (pH 6.0) for ten minutes at 98 °C to reveal to antigen, and subsequently used 5% goat serum for blocking. We applied the primary anti-PgCad1 antibody (final concentration: 4.4 µg/mL) and the secondary goat anti-rabbit fluorescence antibody conjugated with TRITC (final concentration: 2.5 µg/mL) in sequential order to incubate the tissue sections as shown previously^27^. Then, we washed the tissue sections three times using PBS, and performed the fluorescence analysis. The anti-PgCad1 polyclonal antibody was produced by ABclonal Biotechnology Co., Ltd (Wuhan, China) using the purified recombinant protein containing amino acids 22 to 311 (Fig. S1) based on the consensus sequence of the wild and mutant PgCad1 as antigen to immunize rabbit. Two midguts were analyzed for each strain, and three images were viewed for each midgut. We chose the best quality images for each strain for Fig. 4.

### Transcript abundance of *PgCad1* in midgut tissue

We compared transcript abundance of *PgCad1* between APHIS-S and AQ189 using fluorescence quantitative real-time PCR (qPCR). We extracted total RNA from midgut tissue of fourth instar larvae using Trizol (Invitrogen) reagent and detected their quality and quantity using gel electrophoresis and spectrophotometry (Nano-200, Hangzhou Allsheng Instrument Co. Ltd., China). The total RNA (1 µg) from each sample was used to produce first-strand cDNA based on the PrimeScript RT reagent Kit with gDNA Eraser (TAKARA). Two pairs of primers (*PgCad1*qF/*PgCad1*qR, ACF/ACR) were designed for qPCR according to the cDNAs of *PgCad1* (GenBank no. AY198374) and *β-actin* from pink bollworm (GenBank no. MH423440.1). The qPCR for *PgCad1* and *β-actin* genes were conducted on a thermocycler (Eppendorf realplex^4^) using TB Green Premix Ex Taq II (Tli RNaseH Plus) (TAKARA) as follows: 95 °C for 30 s, and then 40 cycles at 95 °C for 5 s, and 60 °C for 40 s, and followed by a melting curve temperature from 60 °C to 95 °C with 0.5 °C intervals. For each sample of larval midgut tissue used for RNA extraction, we pooled midgut tissues from 10 fourth instar larvae. For each strain (APHIS-S and AQ189), we used four samples (total of 40 larvae per strain) and tested each sample three times. For all qPCR assays, we applied a template without cDNA but only sterile water for negative control and quantified the *PgCad1* gene using the *β-actin* reference gene as an endogenous control. We determined the relative expression level of *PgCad1* based on the mathematical models Ct (2^−∆∆Ct^) method as described previously^50^.

### Statistical analysis

We performed probit analyses using SPSS^51^. From the bioassay data, we calculated the LC_50_ (concentration killing 50% of larvae) and its 95% fiducial limits. We also carried out probit analyses of cell toxicity data to determine the EC_50_ (effective concentration causing swelling in 50% of cells) and its 95% fiducial limits. In the genetic linkage analysis, we applied a one-sample t-test to determine if the proportion of *r14r14* individuals surviving on control diet differed significantly from the expected value of 0.50 based on random segregation. To determine if the proportion of survivors that were *r14r14* differed significantly between Cry1Ac-treated diet and control diet, we performed Fisher’s exact test. We carried out one-way ANOVA and Tukey’s HSD test to determine if statistically significant differences occurred in development time or weight of pupae for AQ189 on Bt cotton, AQ189 on non-Bt cotton, and APHIS-S on non-Bt cotton. For AQ189, we applied Fisher’s exact test to evaluate the null hypotheses that survival from neonate to adult and adult sex ratio were similar between Bt cotton and non-Bt cotton.

## Supplementary information

Supplementary file1

Supplementary file2
