# Supplementary material for: Cadherin repeat 5 mutation associated with Bt resistance in a field-derived strain of pink bollworm
Source: Sci Rep. 2020 Oct 8;10:16840. doi: 10.1038/s41598-020-74102-z (PMC7544870; doi:10.1038/s41598-020-74102-z)
Supplement: Supplementary file 1 — Supplementary file1 [file 41598_2020_74102_MOESM1_ESM.docx]

**Cadherin repeat 5 mutation associated with Bt resistance in a field-derived strain of pink bollworm**

**Supplementary Figures S1-S6**

Ling Wang^1,2^, Yuemin Ma^3^, Wei Wei^3^, Peng Wan^1^, Kaiyu Liu^3^, Min Xu^1^, Shengbo Cong^1^, Jintao Wang^1^, Dong Xu^1^, Yutao Xiao^4^, Xianchun Li^5^, Bruce E. Tabashnik^5^ & Kongming Wu^2*^

^1^Key Laboratory of Integrated Pest Management On Crops in Central China, Ministry of Agriculture, Hubei Key Laboratory of Crop Disease, Insect Pests and Weeds Control, Institute of Plant Protection and Soil Fertility, Hubei Academy of Agricultural Sciences, Wuhan, 430064, China.

^2^State Key Laboratory for Biology of Plant Diseases and Insect Pests, Institute of Plant Protection, Chinese Academy of Agricultural Sciences, Beijing, 100193, China.

^3^School of Life Science, Central China Normal University, Wuhan, 430079, China.

^4^Agricultural Genomics Institute at Shenzhen, Chinese Academy of Agricultural Sciences, Shenzhen, 518120, China.

^5^Department of Entomology, University of Arizona, Tucson, Arizona, 85721, USA.

* Correspondence and requests for materials should be addressed to Kongming Wu (E-mail address: [wukongming@caas.cn](mailto:wukongming@caas.cn))


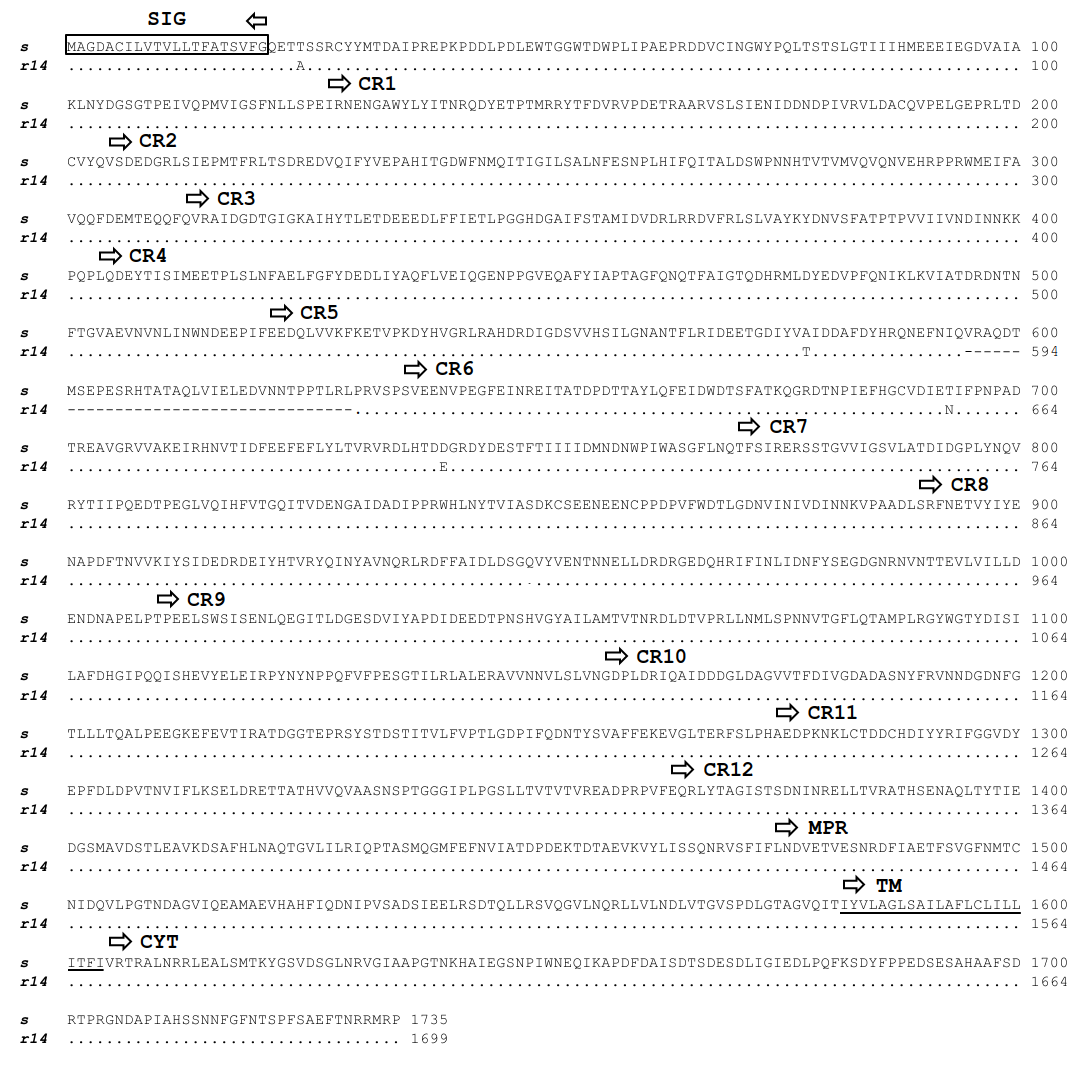


**Figure S1.** Predicted amino acid sequence of pink bollworm cadherin protein PgCad1 for alleles *s* (GenBank accession no. MF276974) from susceptible strain APHIS-S; and *r14* (GenBank accession no. KY814706) from resistant strain AQ189. The *s* allele was sequenced in 2015 from the subset of APHIS-S reared in China [1]. Shown are the signal sequence (SIG), cadherin repeats (CR1-CR12), membrane-proximal region (MPR), transmembrane region (TM) and cytoplasmic region (CYT). The short dashes indicate differences in *r14* compared with *s*: deletion of 36 amino acids (from 595 to 630). The four amino acids in *r14* indicate single amino acid substitutions (T25A, A578T, T693N, D740E). The dots for *r14* indicate the sequence is the same as for the *s* allele.


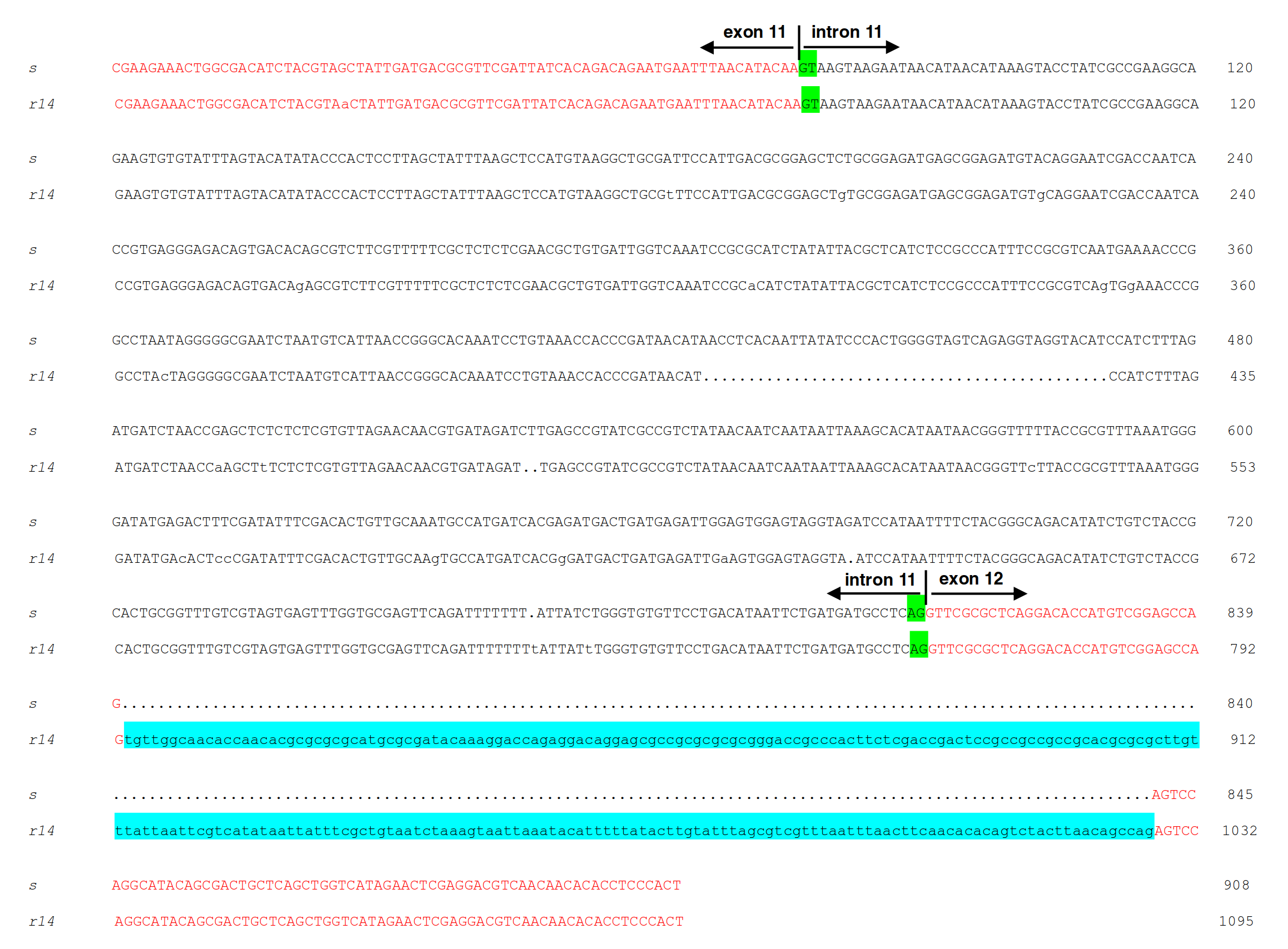


**Figure S2.** Alignment of gDNA sequences of *r14* and *s* alleles. The red letters indicate exon sequences. The letters highlighted in green represent GT/AG splicing sites. The letters highlighted in blue are a 234-bp insertion in exon 12 of *r14*.


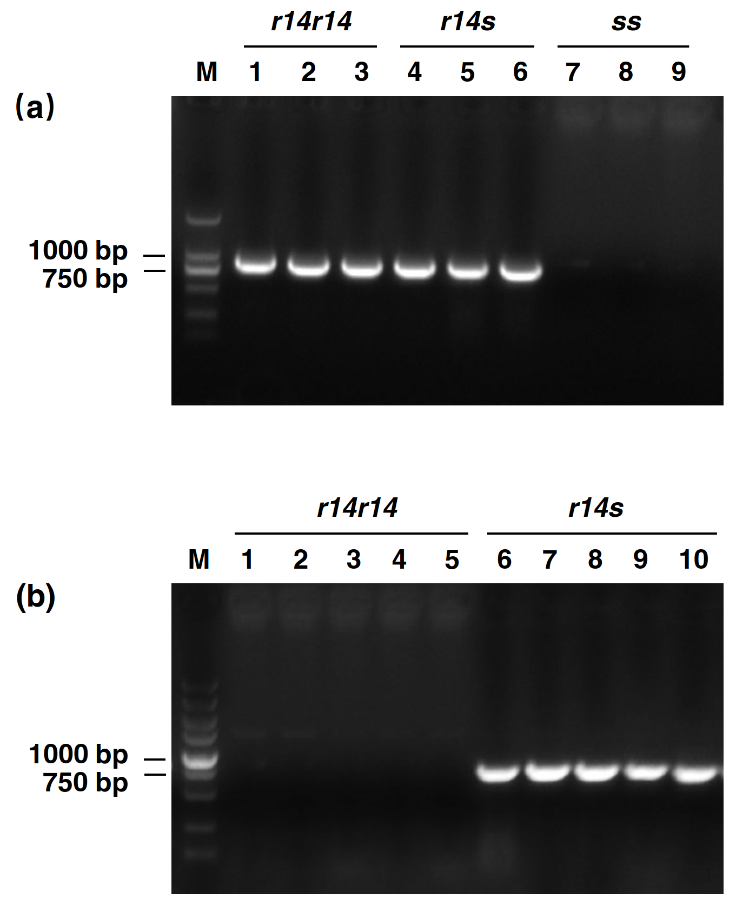


**Figure S3**. PCR detection of *PgCad1* genotype using primers in Table S1. (a) Primers for *r14* (*r14*allF and *r14*R) generate a single band of 852 bp in *r14r14* from AQ189 (lanes 1-3) and in *r14s* (F_1_ progeny of APHIS-S × AQ189; lanes 4-6); and no band in *ss* from APHIS-S (lanes 7-9). (b) Primers for *s* (*r14*allF and not*r14*R) generate no band from *r14r14*(lanes 1-5) and a single band of 853 bp from *r14s* (lanes 7-10)*.*


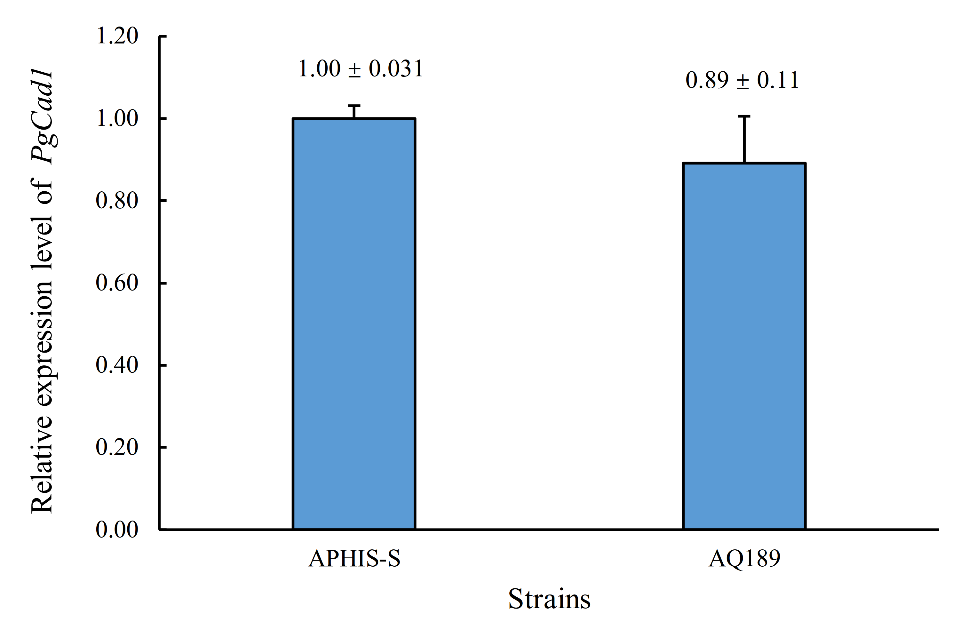


**Figure S4.** Relative transcript levels of *PgCad1* in APHIS-S and AQ189 fourth instar larvae determined by qRT-PCR. Transcript levels of *PgCad1* were adjusted by *β-actin* gene and data above each bar show the mean relative transcript levels of *PgCad1* and the standard errors (SE). Three technical replicates and four biological replicates were used to determine relative transcript levels of *PgCad1* for each strain (n = 4 pools of 10 larvae per strain).


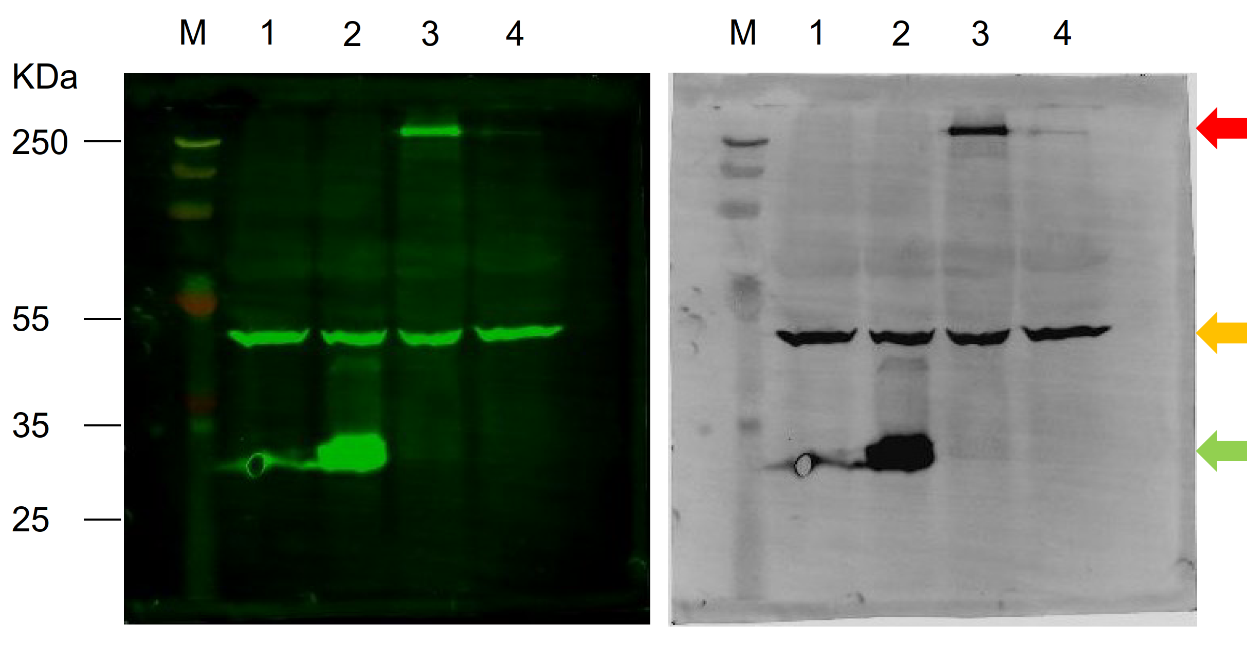


**Figure S5.** Western blots of recombinant cadherin proteins within Hi5 cells. Black and white image at right and color image of the same gel at left. M indicates lanes with molecular weight markers. Blot probed with anti-GFP and anti-actin monoclonal antibodies successively. Protein from Hi5 cells: untransformed cells (lane 1), transfected with the empty vector containing GFP (lane 2), transfected with the coding sequence of sPgCad1-GFP fusion protein with the vector carrying the pink bollworm *s* allele (lane 3) and transfected with the coding sequence of r14PgCad1-GFP fusion protein with the vector carrying the pink bollworm r14 (lane 4). The red, yellow and green arrows point to fusion proteins (sPgCad1-GFP and r14PgCad1-GFP), ß-actin and GFP respectively.**
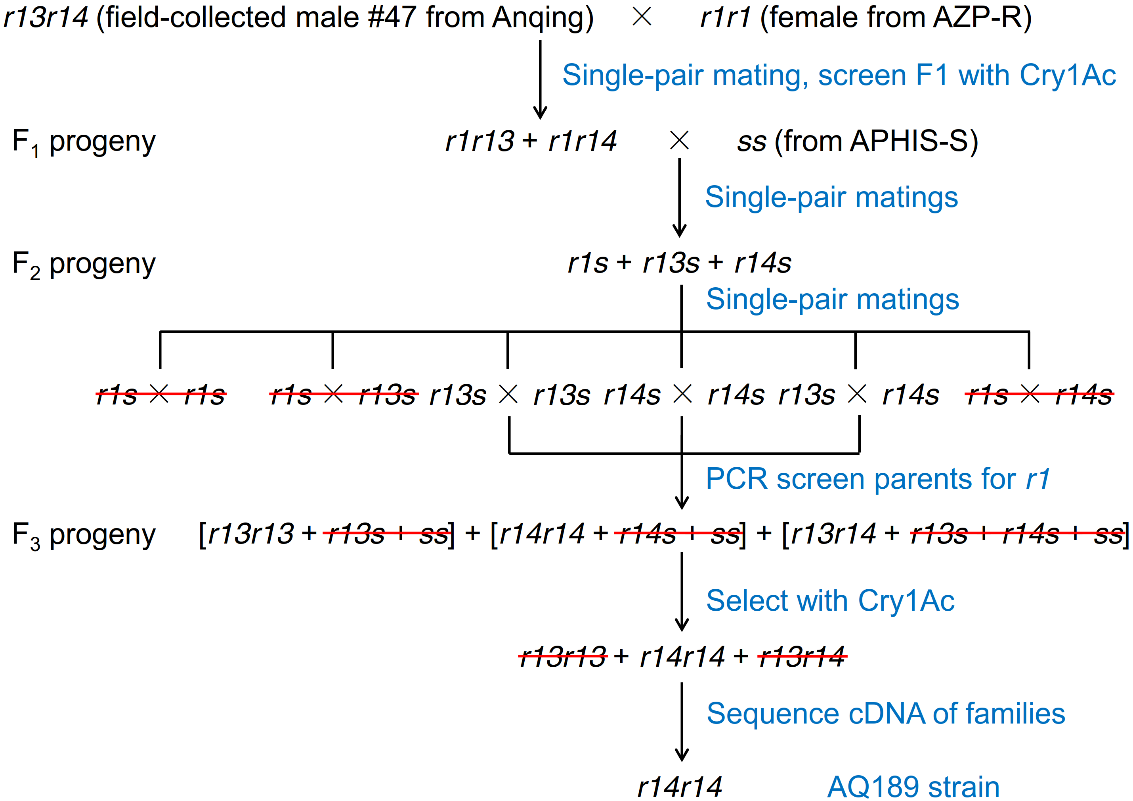
**

**Figure S6.** Isolation of pink bollworm resistant strain AQ189. We started pink bollworm strain AQ189 by pairing a field-collected male (#47) from Anqing in the Yangtze River Valley with a female (*PgCad1* genotype *r1r1*) from the resistant strain from Arizona AZP-R. We screened their F_1_ progeny (family #47) with a diagnostic concentration of Cry1Ac (10 μg Cry1Ac protoxin per ml diet). Based on the 63% survival of the F_1_ offspring at the diagnostic concentration and previously reported recessive resistance to Cry1Ac of pink bollworm [2, 3], we hypothesized that the field-collected male parent of family #47 carried two recessive alleles at *PgCad1* conferring resistance to Cry1Ac. Sequencing of cDNA from the resistant F_1_ offspring revealed that male #47 had two novel mutant cadherin alleles, which we name *r13* [1] and *r14* (Genbank accession numbers KY814706). This paper focuses on *r14*.

To generate resistant strain AQ189, survivors from family #47 were reared to adults and paired individually with adults of the opposite sex from the susceptible strain from Arizona APHIS-S (*ss*). We used PCR to screen the parents of the resulting single-pair F_2_ families for *r1* [4], and retained only the families from pairs of parents that lacked *r1* (*r13s* and *r14s*). After 17 single pairs of these F_2_ progeny were allowed to mate, we selected each of the 17 resulting families (F_3_) separately by exposing larvae to diet containing the diagnostic concentration of Cry1Ac.

After selection with Cry1Ac, we used specific primers (Table S1) to amplify cadherin cDNA from 6 to 8 survivors from each of the F_3_ families. Sequencing of the cadherin cDNA allowed us to group the families into three categories: 1) only one sequence (*r13*) with a deletion of 207 bp (4620-4826); 2) only one sequence (*r14*) with a deletion of 108 bp (1783-1890); or 3) both sequences found in 1) and 2). We pooled all of the survivors from type 2) families (*r14r14*), reared their progeny as strain AQ189, and selected AQ189 every fifth generation by exposing larvae to diet containing the diagnostic concentration of Cry1Ac.

1. Wang, L. *et al*. Resistance to *Bacillus thuringiensis* linked with a cadherin transmembrane mutation affecting cellular trafficking in pink bollworm from China. *Insect Biochem Mol Biol* **94**, 28-35, <https://doi.org/10.1016/j.ibmb.2018.01.004> (2018).

2. Morin, S. *et al*. Three cadherin alleles associated with resistance to *Bacillus thuringiensis* in pink bollworm. *Proc. Natl Acad Sci USA* **100**, 5004-5009, <https://doi.org/10.1073/pnas.0831036100> (2003).

3. Tabashnik, B. E. *et al*. Sustained susceptibility of pink bollworm to Bt cotton in the United States. *GM crops & food* **3**, 194-200, <https://doi.org/10.4161/gmcr.20329> (2012).

4. Morin, S. *et al*. DNA-based detection of Bt resistance alleles in pink bollworm. *Insect Biochem Mol Biol* **34**, 1225-1233, <https://doi.org/10.1016/j.ibmb.2004.08.003> (2004).
