# Supplementary material for: Cadherin repeat 5 mutation associated with Bt resistance in a field-derived strain of pink bollworm
Source: Sci Rep. 2020 Oct 8;10:16840. doi: 10.1038/s41598-020-74102-z (PMC7544870; doi:10.1038/s41598-020-74102-z)
Supplement: Supplementary file 2 — Supplementary file2 [file 41598_2020_74102_MOESM2_ESM.docx]

**Cadherin repeat 5 mutation associated with Bt resistance in a field-derived strain of pink bollworm**

**Supplementary Tables S1-S4**

Ling Wang^1,2^, Yuemin Ma^3^, Wei Wei^3^, Peng Wan^1^, Kaiyu Liu^3^, Min Xu^1^, Shengbo Cong^1^, Jintao Wang^1^, Dong Xu^1^, Yutao Xiao^4^, Xianchun Li^5^, Bruce E. Tabashnik^5^ & Kongming Wu^2*^

^1^Key Laboratory of Integrated Pest Management On Crops in Central China, Ministry of Agriculture, Hubei Key Laboratory of Crop Disease, Insect Pests and Weeds Control, Institute of Plant Protection and Soil Fertility, Hubei Academy of Agricultural Sciences, Wuhan, 430064, China.

^2^State Key Laboratory for Biology of Plant Diseases and Insect Pests, Institute of Plant Protection, Chinese Academy of Agricultural Sciences, Beijing, 100193, China.

^3^School of Life Science, Central China Normal University, Wuhan, 430079, China.

^4^Agricultural Genomics Institute at Shenzhen, Chinese Academy of Agricultural Sciences, Shenzhen, 518120, China.

^5^Department of Entomology, University of Arizona, Tucson, Arizona, 85721, USA.

* Correspondence and requests for materials should be addressed to Kongming Wu (E-mail address: [wukongming@caas.cn](mailto:wukongming@caas.cn))

**Table S1.** Toxicity of Cry2Ab to pink bollworm larvae from AQ189 resistant strain and APHIS-S susceptible strain

| Strain | Slope (SE)^a^ | LC_50_ (95% FL)^b^ | RR^c^ |
| --- | --- | --- | --- |
| APHIS-S | 2.69 (0.339) | 0.157 (0.125-0.188) |  |
| AQ189 | 2.59 (0.357) | 0.286 (0.229-0.343) | 1.8 |

^a^Slope of the concentration-mortality line with its standard error in parentheses.

^b^Concentration killing 50% with 95% fiducial limits in parentheses, in μg Cry2Ab per ml diet.

^c^Resistance ratio, the LC_50_ for AQ189 divided by the LC_50_ for APHIS-S.

**Table S2.** Genetic linkage between resistance to Cry1Ac and *r14*

|  | Larvae with *r14r14* (%) | | Larvae with *r14s* (%) | |
| --- | --- | --- | --- | --- |
| Backcross family | Control diet | Cry1Ac diet | Control diet | Cry1Ac diet |
| 1 | 50 | 100 | 50 | 0 |
| 2 | 55 | 100 | 45 | 0 |
| 3 | 52 | 100 | 48 | 0 |
| 4 | 43 | 100 | 57 | 0 |
| 5 | 44 | 100 | 56 | 0 |
| Mean | 49 | 100 | 51 | 0 |

PCR was used to determine the genotype for a total of 254 larvae: 154 on control diet (30, 31, 31, 30 and 32 from backcross families 1-5, respectively) and 100 on diet treated with the diagnostic concentration of Cry1Ac (20 from each backcross family).

**Table S3.** Survival of AQ189 and APHIS-S larvae on Bt cotton and non-Bt cotton

| Insect strain | Cotton type | Bolls | Entry holes per boll | Survivors/boll | Survival(%) ^a^ | Relative survival (%)^b^ |
| --- | --- | --- | --- | --- | --- | --- |
| AQ189 | Bt | 21 | 6.1 (0.2) | 0.9 (0.1) | 13.9 (1.2) | 48.8 (2.8) |
| APHIS-S | Bt | 35 | 5.6 (0.1) | 0.0 (0.0) | 0.0 (0.0) | 0.0 (0.0) |
| AQ189 | Non-Bt | 18 | 6.2 (0.2) | 1.8 (0.1) | 28.5 (1.4) |  |
| APHIS-S | Non-Bt | 43 | 5.5 (0.2) | 1.7 (0.1) | 31.1 (0.9) |  |

Values are means with their standard errors in parentheses.

^a^Larvae surviving per boll divided by entry holes per boll multiplied by 100%. ^b^Survival on Bt cotton divided by survival on non-Bt cotton.

**Table S4.** Primers used for cloning and genotyping of *PgCad1*

|  | Name^a^ | Primer sequence (5´-3´) | Template | Genotype | Size (bp) |
| --- | --- | --- | --- | --- | --- |
|  | F1 | CATACTGGTGACGGTGCTTCT | cDNA | *ss* | 2384 |
|  | R1 | GGACTTGGTTGTAAAGTGGGC |  | *r14r14* | 2276 |
|  |  |  |  |  |  |
|  | F2 | GACCTTCAGTATTCGGGAGCG | cDNA | *ss* | 2890 |
|  | R2 | CATGCGCCTGTTAGTGAACTC |  | *r14r14* | 2890 |
|  |  |  |  |  |  |
|  | gF189 | CGAAGAAACTGGCGACATCTACG | gDNA | *ss* | 908 |
|  | gR189 | AGTGGGAGGTGTGTTGTTGACG |  | *r14r14* | 1095 |
|  |  |  |  |  |  |
|  | *r14*allF | CGAAGAAACTGGCGACATCTACG | gDNA | *ss* | None |
|  | *r14*R | CGCTCCTGTCCTCTGGTCCTT |  | *r14r14* | 852 |
|  |  |  |  |  |  |
|  | *r14*allF | CGAAGAAACTGGCGACATCTACG | gDNA | *r14s* | 853 |
|  | not*r14*R | GTATGCCTGGACTCAAGCTCCG |  | *r14r14* | None |
|  |  |  |  |  |  |
|  | PgCADF | CCGGAATTCGCCACCATGGCGGGTGA  CGCCTGCAT | cDNA | *ss* | 5205 |
|  | PgCADR | TCCCCGCGGACCGCCTCCGCCACCG  CCTGGTCGCATGCGCCTGTTAGTG | cDNA | *r14r14* | 5097 |
|  |  |  |  |  |  |
|  | *PgCad1*qF | ATCCTCCCCAGTTCGTTTTTCC | cDNA | *ss* | 155 |
|  | *PgCad1*qR | AGTCACCACGCCAGCATCAAG |  | *r14r14* | 155 |
|  |  |  |  |  |  |
|  | ACF | CACCGTGCCCATCTATGAAGG | cDNA | *ss* | 145 |
|  | ACR | GACGATTTCCCTCTCAGCGGT |  | *r14r14* | 145 |

^a^F indicates forward and R reverse.
